# A Qualitative Evaluation of Young People’s, Parents’ and Carers’ Experiences of a National and Specialist CAMHS Dialectical Behaviour Therapy Outpatient Service

**DOI:** 10.3390/ijerph18115927

**Published:** 2021-05-31

**Authors:** Namali Ratnaweera, Katrina Hunt, Jake Camp

**Affiliations:** 1 Department of Psychology, Goldsmiths, University of London, London SE14 6NW, UK; 2 National and Specialist CAMHS DBT Service and Maudsley Centre for Child & Adolescent Eating Disorders, South London & Maudsley NHS Foundation Trust, London SE5 8AZ, UK; Katrina.Hunt@slam.nhs.uk; 3 National and Specialist CAMHS DBT Service, South London & Maudsley NHS Foundation Trust, London SE5 8AZ, UK; Jake.Camp@slam.nhs.uk

**Keywords:** qualitative study, young people, mental health, DBT, BPD

## Abstract

(1) Background: Dialectical Behaviour Therapy (DBT) is the recommended treatment for Borderline Personality Disorder (BPD) symptoms in adults, however, research investigating the effectiveness of DBT for adolescents is limited. The present study explores the experiences of young people and their parents/carers of a DBT service using qualitative methodology. (2) Methods: Young people and their parents/carers, who completed DBT within the National and Specialist Child and Adolescent Mental Health DBT Service based at the Maudsley Hospital in London, were asked questions regarding their experience of the service. Data was collected from young people who completed treatment between July 2019 and July 2020 (*n =* 18) and their parents and carers (*n* = 7). (3) Results: Amongst young people, the themes identified were: a new way of living, better understanding of self, new skills, person-centred approach, and relationships with others. Parent and carer interviews revealed themes of improved relationships, feeling supported, improved quality of life, and time/timing. (4) Conclusions: Young people reported improvements in emerging BPD symptomology after completing DBT. Parents and carers reported improvements in their young person and families since starting DBT. A longer DBT programme, earlier DBT intervention, and the time-consuming nature of DBT were highlighted as areas for improvement.

## 1. Introduction

Adolescence is a critical period in which mental health conditions can emerge. Self-harm and suicide in adolescents are significant public health concerns worldwide [1]. Studies suggest a history of suicide attempts and self-harm are predictors of future suicide attempts and deaths [2]. Research shows that self-harm is a symptom that can precede a diagnosis of Borderline Personality Disorder (BPD)/Emotionally Unstable Personality Disorder (EUPD) and often begins in adolescence [3].

Linehan’s [4] biosocial theory of BPD is a transactional model used to understand the aetiology and pathology of BPD. According to this model, BPD is characterised mainly as a disorder of emotion dysregulation and develops in individuals with a combination of a biological disposition, an objective or subjective invalidating environment, and the transactions between the two during key developmental periods [4]. Linehan’s biosocial theory [4] proposes that individuals with BPD experience problems with regulating many emotions largely due to the predisposed vulnerability to difficult emotions and maladaptive learned emotion modulation strategies.

In addition to self-harming behaviours, individuals diagnosed with BPD can experience patterns of identity disturbance, affective instability, impulsivity, and interpersonal difficulties [5]. Functional and structural deficits have also been identified in areas of the brain such as the frontolimbic system in individuals with BPD [6] and neuropsychological deficits associated with frontal lobe functioning. Neuropsychological research suggests that BPD patients exhibit strong deficits in neuropsychological domains, such as attention, speeded processing, and cognitive flexibility [7].

Epidemiological studies investigating the prevalence of BPD in adolescents suggest various prevalence rates depending on the cohort being studied. For example, within the general population of adolescents, the estimated prevalence of BPD is 3% [8]. However, clinical studies have demonstrated prevalence rates ranging from 11% [9] in outpatient adolescent populations to as high as 78% in suicidal adolescents seen in the emergency department [10]. Individuals diagnosed with BPD have a high prevalence of comorbid conditions, such as depression, anxiety, eating disorders, and substance misuse disorders [11]. This is likely due to the overlap of symptoms that BPD can share with other disorders and the difficulty in distinguishing whether presenting symptoms are a result of BPD, a comorbid condition, or a combination of both within a clinical context [11].

### 1.1. Dialectical Behaviour Therapy (DBT)

DBT is a widely used treatment for BPD symptoms and is outlined in the National Institute for Health and Care Excellence guidelines [12] as the recommended treatment for people with BPD where a reduction in self-harm is the top priority. The treatment goals of DBT include reducing emotional dysregulation in order to reduce other areas of dysregulation, such as self-harm and suicidal behaviours, in relationships, in one’s sense of self, and in unhelpful thinking styles. This is achieved through problem solving strategies, skills training, exposure-based techniques, and principles of validation [4].

### 1.2. DBT for Adolescent Populations

While DBT is demonstrated to be an efficacious intervention for adults diagnosed with BPD, research investigating the efficacy of DBT in adolescent populations is comparatively limited. This lack of research could be accounted for by the hesitancy in diagnosing adolescents with BPD due to adolescence being the time where the personality is still developing, some symptoms characteristic of BPD are normative for this developmental stage, and to shield adolescents from the potential stigma related to the BPD diagnosis [11,13]. Young people who present with persistent diagnostic features of BPD are sometimes referred to as experiencing emerging traits of BPD [14]. Despite this, there has been growing evidence that supports the usefulness of early diagnosis and interventions for BPD in young people such as managing complications of BPD earlier in life and mitigating long-term mental health problems [15].

Much of the research exploring DBT as an intervention for adolescents with emerging BPD are conducted in controlled research contexts such as Randomised Controlled Trials (RCTs). Three RCTs to date have found that compared to individual- and group-therapy control groups, DBT is more efficacious at reducing self-harming behaviours and depression symptoms and increasing global function [16,17,18].

Although RCTs can be extremely valuable in strengthening our understanding of the efficacy of a treatment, it is also important for research to be conducted in routine public health settings.

Studies conducted in public health settings found that adolescents who participated in a DBT programme demonstrated a significant reduction in BPD symptoms, self-harming behaviours, suicidal ideation, and depression as well as a decrease in hospital and emergency department admissions [19,20,21]. Some studies have utilised qualitative methodology to learn about service users’ experience of skills training taught in DBT for adolescents demonstrating the effectiveness of these skills [22]. Though there have been some studies that have evaluated the efficacy of DBT for adolescents in a public health setting and some that have used qualitative methods to explore service user experiences of DBT, there is still a need to widen the qualitative research data for DBT service users in Child and Adolescent Mental Health Services (CAMHS) and their parents/carers.

The aim of the present study is to investigate adolescent service users’ and their parents’/carers’ experiences of a comprehensive outpatient DBT programme at the National and Specialist CAMHS DBT Service in the South London and Maudsley (SLaM) NHS Foundation Trust, using qualitative methodology. The study was conducted as a service evaluation of the DBT service to gain insight into which aspects of the service are successful and what areas can be improved upon.

Since this is qualitative and exploratory research, there are no specific hypotheses being tested. Rather, the following set of research objectives will be examined:

- To evaluate the acceptability of the treatment programme;
- To explore service users’ retrospective expectations of DBT;
- To investigate perceived benefits of the treatment programme, and specifically whether certain treatment components were experienced more favourably and beneficially to service users (e.g., individual therapy, skills groups, and phone support);
- To explore suggestions for improvements and modifications of the treatment programme;
- To explore service user’s experience of using DBT skills to manage their difficulties and investigate the type of skills used outside of therapy; and
- To evaluate service users’ reflections on their symptomatology, problems, and achievements having completed the treatment programme.

## 2. Materials and Methods

Ethical approval was granted by the South London and Maudsley NHS Foundation Trust CAMHS Audit Committee and Goldsmiths, University of London Ethics Committee. Data was collected as part of routine service delivery. Informed consent was obtained for those people participating in the interview.

The study employed a qualitative approach to attain the exploratory aims which were to evaluate experiences of young people and their families of the National and Specialist CAMHS DBT Service. Thematic analysis was selected as the method of analysis as it can allow for an in-depth understanding of the data collected [23]. An inductive approach was used in this analysis as it is a data-driven approach where the themes are strongly associated with the collected data and thus produces a detailed account of the data [23].

The National and Specialist CAMHS DBT Service is a comprehensive community DBT programme based on Linehan’s [4] adult DBT model and Miller, Rathus, and Linehan’s [24] adolescent model, as well as the adapted skills training for adolescents by Rathus and Miller [25]. This eight to 12 month programme is comprised of once-weekly individual therapy sessions, skills coaching delivered over the phone between sessions (Monday to Friday, 9 am to 5 pm) and six months of once-weekly DBT skills groups, as well as a parent/carer skills group that runs concurrently with the young persons’ group and tailored individual sessions for parents/carers.

Young people were accepted into the programme, and thus the study, if they were referred to the service between the ages of thirteen years and seventeen years and two months; if in the past six months they had at least two episodes of self-harm or high risk behaviours; and displayed symptoms in at least five of the diagnostic domains of BPD determined using the Structured Clinical Interview for DSM-IV (SCID-BPD) to assess for symptoms of emerging BPD as well as self-report measures. Young people would not be accepted into the programme and this study if there was a primary diagnosis of another psychiatric disorder needing more urgent assessment or treatment, or if the young person had opted out of the DBT programme in the past three months.

Young people who completed treatment and consented to do an exit interview between July 2019 to July 2020 were included in this study (*n* = 18). The average age of participants was 16.52 years (SD = 1.04) at assessment. Parent/carer interviews were also offered to parents/carers of the young people who completed the programme in this time period. A total of seven parent and carer interviews were conducted with two professional carers and five parents. 

There was a total of twenty-six young people who finished treatment between July 2019 and July 2020. Of these young people, twenty (76.9%) consented to take part in the interviews while six (23.1%) did not complete an interview. Of the twenty young people who consented, two (7.7%) had no audio recordings, leaving eighteen (69.2%) eligible participants for this study.

Interviews were either conducted in-person or via Microsoft Teams (video conferencing software) (Microsoft. Redmond, Washington, USA) by an assistant psychologist working for the National and Specialist CAMHS DBT service.

The interviews consisted of several open-ended questions that assessed service users’ experiences of the service, including their understanding of the DBT service, personal hopes when they came to the service, what they learned from the service, what was helpful/unhelpful about the service, changes they have experienced since entering the programme, how the programme compares to previous therapies they underwent, and advice they would give to future service users’ accessing the service. Participants were prompted and encouraged to expand on their responses. To ensure the participant’s thoughts and feelings were fully understood and accurately reflected, the interviewer summarised participant responses after each question.

Questions asked during the parent/carer interviews differed from the young person interviews as they focused on parent/carer experience of the DBT service and how it impacted their young person and family.

Descriptive statistics of demographic information, scores of BPD symptom measures, and attrition rates are presented. The collected data were qualitatively analysed in accordance with Braun and Clark’s [23] framework for using thematic analysis in psychology.

Before commencing data analysis, the researcher read over the transcriptions of each participant’s interview once, and then read through the interviews again making notes of initial ideas. The latter process was repeated twice to become familiarised with the collected data. Following familiarisation of the data, initial codes were produced and then organised into potential themes. Themes were reviewed by the first author and the two senior authors who worked at the DBT service. The identified themes were reviewed to determine if they were consistent with the coded extracts and all the data. These initial themes were then reviewed again and refined further to produce more specific themes that were properly defined and named, and subsequently arranged into a thematic map. Finally, quotes demonstrating the identified themes were extracted from the interviews.

## 3. Results

### 3.1. Demographics & Descriptive Statistics

The mean age of young people (*n* = 18) was 16.52 years (SD = 1.04; range = 14.3–17.6) years old. In total, 88.9% of young people identified as female, 11.1% as non-binary. Demographic information for young people is presented in Table 1. Demographic information was not collected for parents/carers. Young people included in this sample had an average score of 8.94 (SD = 0.94) with a median of 9.00 on the McLean Screening Instrument for BPD (MSI-BPD) [26]. The young people MSI-BPD scores ranged from 7.00 to 10.00. Parents’/carers’ also scored the MSI-BPD based on their perception of their young person’s BPD symptoms, with a mean score of 7.47 (SD = 2.03) and a median of 8.00. The parent and carer MSI-BPD scores ranged from 3.00 to 10.00. Young people had a mean of 139.50 (SD = 14.45) on the self-report measure, Difficulties in Emotion Regulation Scale (DERS) [27]. There was a median DERS score of 139.00 and the scores ranged from 115.00 to 164.00.

### 3.2. Theme 1: New Way of Living

**Figure 1:**
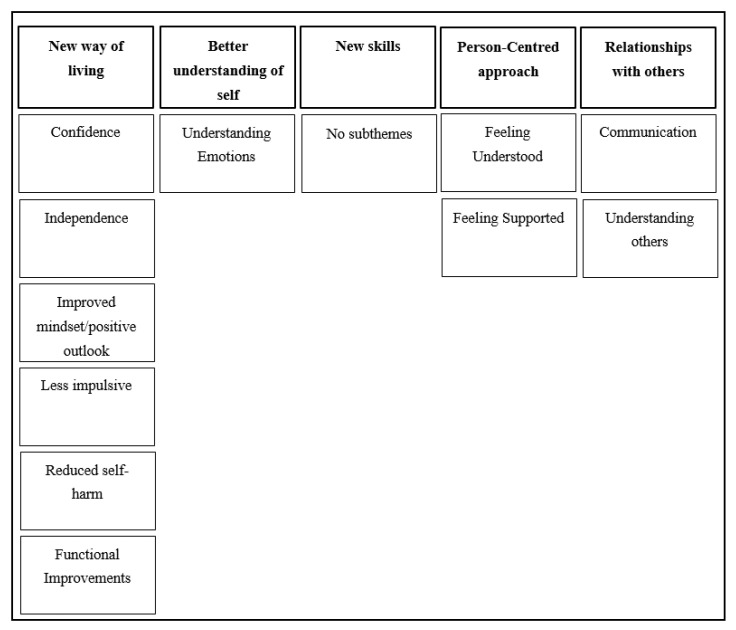
Thematic map representing young person themes and subthemes.

One of the themes that emerged was a *new way of living* (Figure 1). Several participants reported that since they started DBT, they had experienced an improved quality of life.

*P2: I don’t think I maybe have like a quality of life that everyone would think is amazing. But for someone that like is blatant and I’ve got certain difficulties, I think for someone like me, like, wow, my life is so much, so much better.*

#### 3.2.1. Subtheme 1A: Confidence

Within the theme of new way of living, one of the subthemes that emerged was *confidence*. Some participants discussed how they felt, and others had described them as more confident in themselves.

*P6: It was more because like I was not very well, so I kind of, I knew that I seem a lot happier now and that…I definitely seem more confident sometimes…*

#### 3.2.2. Subtheme 1B: Independence

Participants also expressed that through DBT they had developed more *independence*. They found that they were less reliant on others compared to before DBT with some recognising this as a result of help from the therapist and being less anxious.

*P1: And then I think I’m a lot more self-sufficient and before I was too anxious to go out by myself or like do things by myself. And now I’m a lot more willing to do that.*

#### 3.2.3. Subtheme 1C: Improved Mindset/Positive Outlook

Another subtheme of a new way of living was an *improved mindset and increased positive outlook* after DBT. Participants reported that they felt when a difficult situation arose, by the end of DBT, they were better able to manage it due to a difference in the way they think about situations. Some participants also explained how they were able to think more positively, which has influenced their outlook on life.

*P15: I’m less negative about the world, in general, about myself and about others, less judgemental, less comparing, but obviously I still feel like I still do it [judge/compare] ’cause I don’t even notice when I do it half the time. But as soon as I catch myself it’s an opportunity to learn...*

*P14: I can be in a difficult situation and at first be distressed but then think about ways I can help improve it, using like the skills that I’ve been taught, and maybe not just improve the situation but maybe improve my mindset on it instead.*

#### 3.2.4. Subtheme 1D: Less Impulsive

Participants claimed that they had become *less impulsive* since undergoing DBT, by being able to think and waiting to feel calmer before acting in an emotional situation.

*P14: I’m not as impulsive as I used to be. I don’t act straight away in the moment, most of the time. I can calm myself down and think how I can improve the situation in a healthier way.*

#### 3.2.5. Subtheme 1E: Reduced Self-Harm

The third major theme that emerged is *the reduction of self-harm*. Several participants reported that after starting DBT, they experienced a significant improvement in self-harming behaviours and many attributed this to learning skills to manage urges and the attitudes held in DBT that self-harming is a life-threatening behaviour and maladaptive coping strategy.

*P14: Like [before DBT] I got given a few skills to help with self-harm and things like that but there was no kind of reinforcement in it, it wasn’t…seen as such in a negative way. Like if I’d hurt myself, she’d just be like: ‘Oh, okay, what can you do better next time? Let’s carry on.’ Whereas in DBT you’ve got the whole chain thing that you have to do and it’s kind of seen more as a negative thing which in a way I think has helped with me stopping because it’s kind of been reinforced that it’s not a good thing to do compared to before, where it was seen as kind of a little bit normal.*

*P11: I rarely self-harm. I haven’t had a suicidal incident in a long time. I haven’t been in hospital for a very long time.*

#### 3.2.6. Subtheme 1F: Functional Improvements

Participants expressed experiencing *functional improvements* since starting DBT such as now being able to attend school again, being able to spend time with friends, and being able to socialise with people.

*P9: They may have seen like a boost in motivation, considering I was out of school for a very long time probably like almost two years. And then, I’ve successfully integrated from being in hospital to going back to mainstream school.*

### 3.3. Theme 2: Better Understanding of Self

The second major theme to emerge was a *better understanding of self* (Figure 1), an increased appreciation for their own personal interests and identification of their emotions, and how to help effectively manage distress.

*Interviewer: What do these skills and techniques kind of do for you? How do they help you?*

*P10: …they help me to identify what emotion I’m feeling…or where in my body it’s feeling…for example if I’m getting really anxious I’ll get lots of like pain in my stomach or I’ll feel like my chest is really heavy...So instead of like stressing about that I’ll be like thinking “oh, I’m feeling this because of this”, or if I’m feeling really like dissociated or just not connected at all, the grounding techniques really help me to get back.*

*P15: I feel like I thought I knew things before…but I feel like I have a better understanding, like a more broad understanding of just things…that help me.*

*Interviewer: So, is it like a more broad understanding of you or like how to manage…*

*P15: Yeah, just how my mind works, like my thoughts and emotions, like I don’t feel like I’m just a slave to my thoughts and I just react…I’m able now to regain control over that to benefit myself.*

#### Subtheme 2A: Understanding of Emotions

Several participants stated that DBT helped them to build an *understanding of their emotions*, thus allowing them to understand how their emotions influenced other aspects of their life such as relationships, reactions to different situations, and communication. Participants also described improved insight into the cause of difficult emotions and how to identify what they are feeling.

*P18: I’m much more aware of my thoughts and of like where they’re coming from, and whether they are rational or not. I therefore find it easier to control my behaviour…I find it easier to regulate emotions.*

*P1: I think it gave you a chance to really explore your feelings on why you felt this way and I think it was different to a lot of things I’ve done in the past...It’s more specific and you look into your traumas more in depth. And look at them as reasons for emotional responses and I thought that was really useful and it gives you insight into why you react in certain ways so you can then control the way you react.*

### 3.4. Theme 3: New Skills

*New skills* (Figure 1) were reported to have helped with understanding different emotions and improving quality of life and relationships. Participants also highlighted the approach of DBT as being more solution focused, which has equipped them with applicable skills to help when distressed.

*P2: …felt like I was just a slave to my emotions and now having those skills and having like everything I learned in group…I actually feel like I have a functioning life now.*

### 3.5. Theme 4: Person-Centred Approach

Participants attributed one of the useful qualities of DBT to the *person-centred approach* (Figure 1) towards the young person and their needs. Participants felt that though DBT was a structured programme, it was still effectively tailored to their needs and offered flexibility, which was seen as a positive compared to other past therapies they had received.

*P10: …like other therapies that I’ve had, they’re all quite broad, so the therapies will be exactly the same for every individual while like I feel like DBT can be very specified for that person even though it’s the same DBT…so I think that’s why it’s very beneficial.*

#### 3.5.1. Subtheme 4A: Feeling Understood

Within the theme of person-centred approach, *feeling understood* emerged as a subtheme. Some participants felt that the DBT groups components were helpful as it allowed them to meet other young people who had similar experiences and thus allowed them to feel understood and less alone.

*P3: I found group quite helpful because…it makes you feel like you’re not alone. There are other people actually there and know how you feel.*

Other participants reported that at times they could have felt better understood by his or her therapist.

*P3: That staff need to understand they need to…just they can’t assume things…just ask your patient or client…you may have assumptions but don’t tell them until like the reason they done something has come out and just like ‘well, this is what I thought, but I’m glad you kind of told me it wasn’t that’. That’s how I think it should come.*

#### 3.5.2. Subtheme 4B: Feeling Supported

Another subtheme of *person-centred approach* was *feeling supported*. The relationship participants had with their individual therapist and the DBT team made them feel supported.

*P16: I think the support. Like you have to have a good therapist, you have to have a good care co-ordinator, you have to have a good team, to use the skills. And when I was ever stuck on a skill or I didn’t know like what to do or I was just distressed, they would help me get myself out of that…*

Conversely, some participants felt unsupported when accessing the phone coaching aspect of DBT, especially when the 24-h rule was in place as they felt that was a time when they needed more support than usual.

*P14: …there are some times when I have got frustrated because of phone coaching. And maybe that’s kind of down to my thought process instead of actually DBT. I don’t know, but when I’m looking for kind of reassurance on something and I get told to use skills…I know it’s phone coaching for skills but it’s a bit invalidating sometimes?*

### 3.6. Theme 5: Relationships with Others

Participants explained how DBT had influenced the way they behave in their *relationships with others* (Figure 1). Some participants discussed closer relationships with family and friends, and some explained how they were better able to maintain relationships that were previously difficult.

*P3: One of my relationships has improved and that’s with my mum…I think it was more that [the therapist] kind of explained a couple of things to my mum that I don’t think she understood before.*

#### 3.6.1. Subtheme 5A: Communication

Many participants explained how they had developed improved *communication* skills which led to an increase in their ability to articulate their feelings and thoughts, apologise, and being able to communicate openly which improved their relationships with others.

*P18: …definitely my behaviour towards relationships because before I would never be able to say sorry. I would just literally go at people, and say whatever I wanted to say. Now, I realise I have to say sorry.*

One participant felt that during times of crisis, or in some therapy sessions, that communication from their therapist involved asking too many questions.

*P18: … [the therapist] could improve in terms of knowing when to stop asking questions. Sometimes [the therapist]…asks the same questions again and again...Or if it’s at a time when I can’t answer for a reason, like I’m really dissociated… [the therapist] still asks the question, like “what do you need?”…again and again. But if I’m not in the place to answer that and I say like ‘I can’t answer this right now’, [the therapist] just keeps asking it for too long, [the therapist] doesn’t know when to stop.*

#### 3.6.2. Subtheme 5B: Understanding Others

Participants stated that they had an improved *understanding of others* which contributed to improvements in their relationships. Participants attributed this to the skills learned in DBT that allowed them to see other people’s perspectives, validate others, and care for others.

*P18: I feel much more able to…communicate with people in the right way, in terms of like validating them, understanding their needs, and also communicating when I need help.*

### 3.7. Theme 1: Improved Relationships

**Figure 2:**
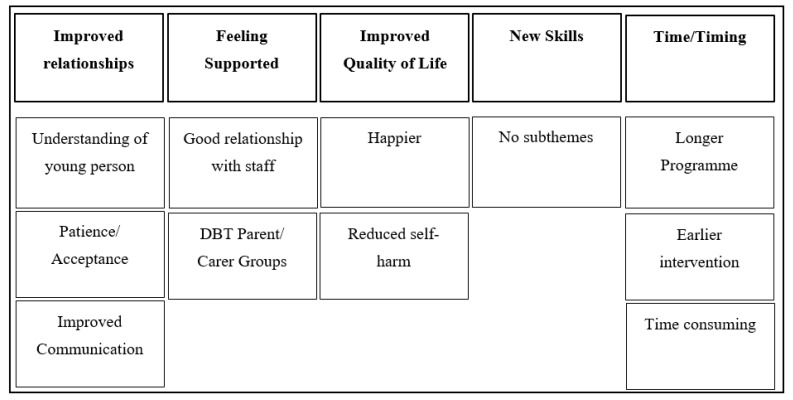
Thematic map representing parent and carer themes and subthemes.

Parents and carers reported that by the end of DBT they felt closer with their young person due to having a better understanding of his or her needs and an *improvement in their relationship* (Figure 2) with their partners as they learned how to support each other and communicate better when they needed help.

*P4: Closer relationship. I think I would say it’s helped with, specially with her dad ’cause they were not as close. I think him coming along and being part of it, too, and learning things has helped him to understand so their relationship [has] improved.*

*P6: …realising that actually we are human so me and my wife are working as a team...recognising when my wife might need some help and recognising…when I might not be so great at communicating…helping me out in those situations.*

#### 3.7.1. Subtheme 1A: Understanding of Young Person

Subtheme 1 from the parent and carer sample represented gaining a better *understanding of the young person*. This was reported amongst all the parents and carers and was suggested to be one of their key hopes at the beginning of the service which they felt was achieved through DBT.

*P4: The understanding of why she feels how she feels and like for me coming to…the parent meetings and being, you know, more understanding. And, you know, having the understanding of why they’re doing what they’re doing and why their emotions are up and down. You know, breaking down what is happening and understanding why they’re doing it.*

#### 3.7.2. Subtheme 1B: Patience/Acceptance

*Patience and acceptance* of the situation was reported to have contributed to parents’/carers’ better understanding of the young person. Parents were able to understand that the young person’s recovery is a continual work-in-progress and were better able to accept the situation they were in.

*P16: Yeah, I think it’s just to look at life in a different way…to be more accepting. More accepting of the good and bad as well and just accepting of the situation. This is…what it is, we’re gonna try and make the best of it and, obviously, if it improves, it’s a blast, but at the moment we work with what we’ve got and we’ll have to give it our all and we’ll see, it could be worse. We were at a worse place [before].*

#### 3.7.3. Subtheme 1C: Improved Communication

*Improved communication* was suggested as one of the reasons for improved relationships between parents/carers and their young person. Parents and carers felt they were taught how to effectively communicate with the young person and with each other. They described how the young person was able to be more open and articulate how they were feeling, which helped parents and carers to understand how to best help them.

*P3: [The young person] is opening up to me a lot and…(inaudible) my other member of staff…[the young person] is very open talking about [the young person’s] emotions and the way [the young person] feels at times when…[the young person] was a bit reluctant in the beginning. But, at the end of the day, I think again the credit goes to [the therapist]. The way, the things have been explained, we have been told how to communicate with [the young person], how to deal with situations. [The young person] has improved in applying DBT skills, regulating emotions and then communicating with the staff has improved more.*

*P4: The last couple of years has been absolutely awful…I’m sure we’re not the first parents that you get a kid who’s self-harming, a kid who wants to kill themselves and you look at each other and you start picking at bones at each other and it drives a big wedge between the two of you…and the communication breaks down between the two of you and, yeah, that’s what DBT is about, communication, and I think it has helped us as well, not just [the young person], I think it’s helped us.*

### 3.8. Theme 2: Feeling Supported

Parents and carers reported that the service was very *supportive* (Figure 2) of the needs of the young person and their families. Parents felt that themselves and their young person had access to support when it was required, and the support was provided by DBT staff who understood the young person’s needs.

*P3: The only advice that I would give the families who were starting…DBT…is that they’re in the most safe hands. And they should engage really well in all of the sessions, with all the professionals and I’m sure they will be looked after very well*.

#### 3.8.1. Subtheme 2A: Good Relationship with Staff

Parents and carers felt that both them and the young people had a good support system and *good working relationship with the staff*. Carers expressed that they had a strong working relationship with the DBT staff that helped them to understand how to support the young person in their care. Parents felt it was useful to have a team of staff to help where the young person had their own therapist and parent/carers had their own separate worker.

*P1: …the biggest thing that I thought [the young person] benefitted from was the one-to-one relationship with [the therapist]…with the previous services we had, if it got down to discussing difficult situations, as soon as…the staff started trying to take a more neutral stance, trying to support us a bit as parents, it felt like to [the young person] that she was being picked on or we were ganging up on her. Whereas [the therapist] was able to maintain…the kind of closer relationship with [the young person] throughout and…that really encouraged her, and I think really helped.*

#### 3.8.2. Subtheme 2B: DBT Parent/Carer Groups

*DBT parent and carer groups* were viewed as providing a supportive environment for parents and carers where they could share their experiences. Parents explained how the groups made them feel understood and less alone in their situation because it allowed them to meet others going through similar problems. It was also highlighted that the parent/carer groups helped lessen feelings of guilt or thoughts about not being a good enough parent.

*P5: I think I really needed the parent group at the time…it’s nice to know that you are not the only one dealing with it…it makes such a difference to think that it’s not just you and…it’s normal parents…you just feel like a terrible person sometimes and there’s other people in there that are just nice people. Not all these horrible people that because you think ‘Am I a terrible person?’…they’re just normal parents living their everyday life, having struggled with their children*.

### 3.9. Theme 3: Improved Quality of Life

Parents and carers expressed that both the young person and their families have an *improved quality of life* (Figure 2) since finishing DBT. Parents found they can manage distressing situations better and understand what they can do to help their young person. One carer noted that their young person is now able to do things on his or her own that they were once unable to do, such as going shopping.

*P2: And we’ve just been doing like normal things, like taking her shopping, that’s been amazing. Where, to start with, we couldn’t leave her in a shop on her own, we had to stay with her, she didn’t feel strong enough to just stand in a shop and you know browse. We had to be like two centimetres away from her.*

#### 3.9.1. Subtheme 3A: Happier

One of the subthemes of improved quality of life was that families and their young person reported feeling *happier* since starting DBT. Parents and carers stated that their general family life was happier than before and homelife had improved. One carer stated their young person was much happier and felt reassured through DBT.

*P6: …generally that we’re a happier bunch than maybe we were before. So, yeah, there’s a strength as well.*

#### 3.9.2. Subtheme 3B: Reduced Self-Harm

Several parents and carers described a *reduction in self-harm*, suicidal ideation, and Accident & Emergency (A&E) visits for their young person, which contributed to an improved quality of life. One carer explained how this has helped their young person to be calmer.

*P2: …she’s not had any self-harming, any major incidents for quite a while now…things have been a lot calmer at home since she’s not being having to go to A&E.*

### 3.10. Theme 4: New Skills

Both parents and carers discussed how learning *new skills* (Figure 2) was very useful for both the young person and his or her families. Parents found that skills helped the young person to manage distress better and that young people were able to apply these skills into their daily lives which helped with managing risky behaviours.

*P5: She knows how to deal with things so much better because of all the strategies she’s learned through the DBT treatment, and when she’s getting stressed with something, she uses these.*

The skills were also helpful for the parents themselves and they were able to incorporate it into different aspects of their lives, such as work and sleep:

*P4: Even the…mindfulness and relaxation techniques, I never thought that I’d embrace it and there I am downloading my apps…*

### 3.11. Theme 5: Timing/Time

*Timing and time* (Figure 2) were considered by all parents and carers as an area of improvement for the service. One parent found it difficult to attend meetings when given last-minute notice and meant that she had to miss important meetings.

*P5: …not so helpful was probably…phone calls last minute for a meeting and expecting to drop things at the last minute…And I’m a single parent that works and somebody needs to make money and I can’t just drop things…Yeah, and as much as I want to go and be part of the meeting, I can’t just drop things just like that because if I drop things just like that I won’t get paid.*

#### 3.11.1. Subtheme 5A: Longer Programme

Several parents and carers suggested that the programme may benefit from being longer as they felt their young person would have benefitted from further DBT. Both carers and some parents expressed that their young person had wanted to continue for longer due to the relationship with his or her therapist and due to future anticipated needs.

*P2: …it’s only time related, it’s only a year. And I know that it’s expensive, but for [the young person] it would’ve been better for her to go on a little bit longer, to possibly extend it…particularly for [the young person], she’s got a difficult time around [date] next year and it would’ve been beneficial to her to have the extra support in place…we knew it was a year when she started, but that’s still not helpful to her.*

#### 3.11.2. Subtheme 5B: Earlier Intervention

Some parents felt that DBT would have been more helpful if their young person had been referred to the DBT programme a few years earlier.

*P5: …I wish we had this service a good couple of years before. We probably wouldn’t have had all the emotional upheaval that we have had, I think it would have been a lot better. And maybe [the young person] wouldn’t have cut as much as she had done and in the emotional state, she had been in…*

#### 3.11.3. Subtheme 5C: Time-Consuming

Many of the parents and carers discussed the *time-consuming* nature of DBT as it was a year-long intensive programme. Some parents reported that they were happy to engage with the service and attend parent/carer sessions, but they had to make special arrangements with employers to achieve this. They noted that this could be difficult for single parents or working parents who cannot make arrangements with work.

*P1: Obviously, it takes a lot of time, for the best part of a year. I had to get special permission from my employers so that I could take time off…luckily, I’m in a job where I was able to get that permission, but…I didn’t miss a session…and neither did my wife. But I know…that could be a problem. It wasn’t a problem for me in the end, but I had sacrificed a promotion to basically to be able to do this but for me it wasn’t really a decision because you know obviously it’s for [the young person’s] benefit and ours.*

## 4. Discussion

This study evaluated the experiences of young people and their parents/carers who had finished a comprehensive outpatient DBT programme at the National and Specialist CAMHS DBT service. The aim of this study was to explore what features of the service were helpful and unhelpful using qualitative methodology. A major theme that emerged amongst young people was a *new way of living* which included subthemes of *confidence*, *independence*, *an improved mindset/positive outlook*, *less impulsivity*, *reduced self-harm*, *and functional improvements.* They expressed how they were able to gain a *better understanding of self* which was attributed to developing a *better understanding of their emotions*. Another theme was the *person-centred approach of DBT* which had subthemes of *feeling understood and supported*. Additionally, young people discussed the influence DBT had in improving their *understanding of others and communication*, subthemes that led to the emergence of another major theme, *relationships with others.*

Parents and carers found that they had *improved relationships* with both their young person and their partners and attributed this to subthemes of gaining a better *understanding of the young person*, *patience/acceptance*, and *improved communication*. Another theme that emerged was feeling supported during their young person’s time with the DBT service as a result of the *good relationship with staff* and participating in the *DBT parent/carer groups.* Parents and carers reported a major theme of *improved quality of life* and described being *happier* and observing *reduced self-harm* in their young person. The improvements that parents and carers suggested for the DBT service were related to the theme of *time/timing* with subthemes of a *longer programme* and *earlier intervention* suggested as being more beneficial, and the subtheme of the *time-consuming* nature of the programme posing difficulties for single and working parents.

The findings of the present study were consistent with the findings of quantitative studies that have investigated the efficacy of DBT for adolescents. For instance, self-reported reduction in self-harming and suicidal ideation in the present study are similar to RCTs examining effectiveness of DBT [17] and to studies in public health settings [21]. Furthermore, self-reported improvements in BPD symptomology are in accordance with the primary targets of DBT. For example, one of the targets is increasing behavioural skills which are used to help with management of BPD symptoms [4]. Amongst both young people and parents/carers, improved relationships and better understanding of the young person were reported. Many participants attributed these improvements to learning the different skills and applying them in various aspects of their lives. These findings are similar to Pardo et al.’s [28] study that found that adolescents completing a DBT skills training group considered DBT skills to be advantageous in helping them with interpersonal relationships and managing distressing situations. Moreover, participants reported developing skills in DBT that aided with controlling impulses, managing emotions, and identifying and understanding emotions resulting in improved emotional regulation. Emotional dysregulation is the fundamental characteristic of BPD that DBT aims to treat [4], thus these findings provide further evidence for the effectiveness of DBT in treating individuals with BPD.

The present study found parents and carers, and young people felt largely understood and listened to by the DBT staff which contributed to an improved understanding of the young person’s needs and learning how to best support the young person. However, some participants reported that at times when they felt less understood by their therapist, this was unhelpful.

### 4.1. Implications

The self-reported reduction of BPD symptomology in young people is suggestive of positive future implications related to their mental health and access to healthcare services and thus support the notion of early intervention for young people with emerging BPD symptomology. Prospective data in the Children in the Community Study suggested that experiencing an increased level of BPD symptomology in adolescence can have a long-term adverse impact on their mental health outcomes in the future [29]. Additionally, in the transitional period into young adulthood, BPD symptoms can lead to romantic dysfunction such as unwanted pregnancy and partner discontentment [30]. Research has suggested the role early intervention can play in mitigating these potential problems [31] and can be considered a cost-effective intervention for this population in the long-term [32].

Implications for the service and services like this is the insight into service user experiences of what works and what works less well. Suggested improvements for the service included considering referrers referring young people to the service earlier, availability of longer treatment length, more notice given to parents and carers to make arrangements to attend meetings, and consideration of the time involvement implications for parents/carers. These suggestions could lead to the opportunity for service improvements in these areas.

### 4.2. Strengths and Limitations

A key strength of this study is that it provides further research to represent the voices and experiences of service users in a DBT programme for adolescents. Similarly, another strength of this study is the inclusion of parent and carer interviews to evaluate their experience of the DBT service, which is not known to have been done in previous studies on general DBT programmes for adolescents with emerging BPD symptoms. Research that has included parent/carer experiences were studies looking at DBT for young people with eating disorders, oppositional defiant disorder, and intellectual disabilities [33,34,35]. While the present study provides preliminary insight into the experiences of parents and carers, it included a small sample size of only five parents and two carers. The small sample size included in this study could be explained by high demands on parents and carers and, thus, difficulty providing additional time along with the expected parent/carer time commitment to the DBT service, resulting in fewer parents and carers being able to commit to participating in the exit interviews. Therefore, similar future studies should aim to continue to include parents and carers to address the paucity in research.

Another limitation of this study is that the sample of young people was made up of only those assigned female sex at birth, and thus did not include any natal male participants. While studies have found that there is no gender difference in the prevalence of BPD [36], sampling bias and gender differences in clinical presentation of BPD could account for the overrepresentation of females with BPD in research [37]. The sampling bias in this paper is similar to other research examining gender differences in BPD which have found that females with BPD symptomology are more likely to seek help through mental health services, including pharmacotherapy and psychotherapy, whereas males with BPD symptomology are more likely to access substance-abuse treatment services [37,38].

The study did demonstrate that participants experienced DBT as an effective intervention in reducing BPD symptomology and improving quality of life in adolescents with BPD symptomology. Whether the improved outcomes were sustained after treatment remains unknown since data were collected at the end of treatment and not followed up afterwards. Few studies have investigated long-term effects of younger populations who undergo DBT, as outcomes were only reviewed at end of treatment or a year after completion [19,39]. Therefore, it would be useful to follow up with these patients to determine if these effects are long-lasting after discharge.

## 5. Conclusions

This study explores the experiences of young people and their parents and carers of a DBT service for adolescents in a UK public health setting. This evaluation demonstrated the effectiveness of DBT as young people and parents/carers reported an improved quality of life and improvements in BPD symptomology. Time involved in the programme and timing of the programme were highlighted as possible areas of improvement, with parents/carers reporting that they would have liked their young person to be referred to the service earlier. Findings from this study provide implications for further research exploring parent/carer experiences of DBT service, follow-up studies to determine sustainability of DBT outcomes and service improvements.

## Figures and Tables

**Table 1:** Young Person Demographics.

Factor		Total Sample (Percentage)
Gender Identity		18 (100%)
	Female	16 (88.9%)
	Non-Binary	2 (11.1%)
Sex Assigned at Birth *	Female	18 (100%)
Sex Assigned at Birth Same or Different	Same	16 (88.9%)
	Different	2 (11.1%)
Sexual Orientation	Heterosexual	8 (44.4%)
	Bisexual	2 (11.1%)
	Gay or Lesbian	1 (5.6%)
	Demisexual	1 (5.6%)
	Queer	1 (5.6%)
	Pansexual	1 (5.6%)
	Asexual	1 (5.6%)
	Prefer not to say	2 (11.1%)
	Unknown	1 (5.6%)
Ethnicity	White (White British, White Other)	11 (61.1%)
	Black African, Caribbean, or Black British	1 (5.6%)
	Mixed or Multiple Ethnic Groups	4 (22.2%)
	Other White/Mixed European	1 (5.6%)
	Other Ethnic Group (Hispanic)	1 (5.6%)

* Sex assigned at birth is same or different to current gender identity.

## Data Availability

The data are not publicly available or available on request due to being collected as part of routine clinical practice.
